# The prevalence of major depression and generalized anxiety disorder in patients with multiple sclerosis in Saudi Arabia: a cross-sectional multicentered study

**DOI:** 10.3389/fpsyt.2023.1195101

**Published:** 2023-08-29

**Authors:** Anas M. Alswat, Bsaim A. Altirkistani, Anas R. Alserihi, Osama K. Baeshen, Eythar S. Alrushid, Joud Alkhudair, Abdulaziz A. Aldbas, Osama M. Wadaan, Ahmad Alsaleh, Yaser M. Al Malik, Ahmad A. Abulaban, Seraj Makkawi

**Affiliations:** ^1^College of Medicine, King Saud bin Abdulaziz University for Health Sciences, Jeddah, Saudi Arabia; ^2^King Abdullah International Medical Research Center, Jeddah, Saudi Arabia; ^3^College of Medicine, King Saud bin Abdulaziz University for Health Sciences, Riyadh, Saudi Arabia; ^4^Division of Neurology, King Abdulaziz Medical City, Ministry of the National Guard Health Affairs, Riyadh, Saudi Arabia; ^5^Department of Medicine, King Abdulaziz Medical City, Ministry of the National Guard Health Affairs, Jeddah, Saudi Arabia; ^6^King Abdullah International Medical Research Center, Riyadh, Saudi Arabia

**Keywords:** multiple sclerosis, autoimmune disease, central nervous system, psychiatric disorder, generalized anxiety disorder, major depression

## Abstract

**Background:**

Multiple sclerosis (MS) is an autoimmune disease characterized by chronic, progressive neurodegeneration of the central nervous system (CNS), and it is the most common inflammatory neurological disease affecting young adults. Given the chronic, progressive nature of the disease, psychiatric disorders are more prevalent among these patients, as reported in the literature; however, data in Saudi Arabia are limited. This study aimed to estimate the prevalence of major depression and generalized anxiety disorder in patients with MS and their association with different patient demographics.

**Methods:**

This was a cross-sectional, multicentered study that included adult patients with MS from 30 June 2021 to 30 June 2022. Participants were interviewed in person and asked to complete a survey that included general demographics, the Patient Health Questionnaire-9 (PHQ-9), and the Generalized Anxiety Disorder-7 (GAD-7) questionnaire. Other variables related to the patients' conditions, such as MS type and Expanded Disability Status Scale (EDSS) score, were collected from the patient's electronic records. Descriptive statistics were performed, and associations were made using the chi-square, Fisher's exact, and analysis of variance tests, as appropriate.

**Results:**

A total of 192 participants were included in this study. Based on a cutoff score of >10 on the GAD-7 and PHQ-9 scales, the prevalence of generalized anxiety disorder was 26.1% (50), with the majority of participants having minimal anxiety (40%); meanwhile, the prevalence of major depression was 42.7% (*n* = 82), and most of them had mild depression (30%). Female participants scored significantly higher compared to men on the GAD-7 scale (*p* = 0.0376), but not on the PHQ-9 scale (*p* = 0.1134). In addition, no statistically significant association was detected between functional disability (EDSS score) and prevalence of anxiety and depression.

**Conclusion:**

This study demonstrated a high prevalence of generalized anxiety disorder and major depression among patients with MS compared with that in the general population, with women being more affected. As these comorbid disorders could negatively affect the disease course, screening is of paramount significance.

## 1. Introduction

Multiple sclerosis (MS) is a chronic, progressive neurodegenerative autoimmune disease that mainly affects the central nervous system (CNS) (1). The main characteristics of MS are demyelination and inflammation of the myelin sheath, which covers the axons of the CNS cells (1). MS is characterized by multiple different subtypes, with relapsing–remitting multiple sclerosis (RRMS) being the most commonly reported subtype (2). The clinical manifestations of MS vary as the patient's presentation depends mainly on the location of the lesion within the CNS, which can occur in the brain, spinal cord, or optic nerves. However, the most common clinical features reported are impaired vision, weakness, numbness, cognitive and psychiatric symptoms, and bowel or bladder dysfunction (3, 4).

MS is the most common inflammatory neurological disease in young adults, affecting more than 2.3 million people worldwide, and the prevalence of the disease has continued to increase since 1990. In Saudi Arabia, the estimated prevalence of MS was ~40.40 per 100,000 population and 61.95 per 100,000 Saudi nationals in 2018 (3). It is more common among women and usually occurs between the ages of 20 and 40 years (4).

MS is considered one of the leading causes of neurological non-traumatic disability among young people, resulting in a reduction in productivity and quality of life and major psychological and social impairment. As such, the prevalence of psychiatric disorders among these patients is higher. Psychiatric manifestations include, but are not limited to, depression, anxiety, mania, and substance use disorders (5–7). Depression is considered the most commonly reported psychiatric disorder among patients with MS (37–54%). This is followed by anxiety disorders (14–41%) (6, 8). In addition, psychiatric manifestations could be reported as the presenting symptom of MS in some patients; however, they appear later in the course of the disease in the majority of patients with MS. The causes of developing these psychiatric comorbidities may be immunological or biological, or due to poor coping mechanisms (9). More importantly, the effects of the disease itself on the brain, such as neuroinflammation, synapses dysfunction, and other structural changes such as hippocampal shape variation and progressive gray matter loss in limbic basal ganglia, may play a role in the pathogenesis of these disorders (10, 11).

Several studies have evaluated the prevalence of psychiatric disorders in patients with MS. In a study published in 2015 to assess the burden of psychiatric diseases (e.g., depression, anxiety, bipolar disorder, and schizophrenia) among patients with MS in comparison with the general population, the prevalence and incidence of the psychiatric disorders mentioned earlier increased among patients with MS compared with those in the general population (12, 13). In this study, several factors were found to be associated with a higher percentage of depression among patients with MS, including female sex and older age (13). Furthermore, Beiske et al. found that fatigue and being young at the time of diagnosis are linked to the development of depression and anxiety symptoms (14, 15). Locally, a study in Saudi Arabia aimed to assess the prevalence of depression in patients with MS and found that 89.9% of patients had mild to severe symptoms of depression (16). Among the patients with depression, 65.13% were non-smokers, and 37.39% were unemployed (16). An association between educational level and severity of depression was also found (16).

Although several studies have evaluated psychiatric diseases among patients with MS, data in Saudi Arabia are limited, and the other studies were survey-based without direct clinical patients' evaluation. Therefore, the focus of this study was to estimate the prevalence of major depression and generalized anxiety disorder in patients with MS in Saudi Arabia and also to find its association with multiple demographic variables, such as age, sex, marital status, educational level, and monthly income, as well as the effects of duration of the disease on patients' mental health. In addition, we compared MS disease-modifying therapy types and severity of the illness using the clinically confirmed EDSS score of the patient with the development of psychiatric comorbidities.

## 2. Materials and methods

### 2.1. Study design and participants

A cross-sectional study with retrospective chart review was conducted at King Abdulaziz Medical City (KAMC) in Jeddah and Riyadh, Saudi Arabia, from 30 June 2021 to 30 June 2022. The inclusion criteria were a confirmed diagnosis of MS based on the McDonald criteria and an age of >18 years. To ensure the quality of the data given, patients were interviewed in person upon attending the neurology clinic and given a survey to fill out. Then, the electronic records of prospective patients were reviewed to complete the data collection process. Informed consent was obtained from each patient to participate in the study.

### 2.2. Study measures

Participants' demographic data, such as age, sex, marital status, monthly income, employment status, history of psychiatric disorders, or any other comorbidities (e.g., diabetes mellitus, hypertension, and chronic kidney disease), were collected. Data related to the patient's condition, such as MS type, EDSS score, type of MS medications, and any other medications (e.g., antidepressant, anxiolytic, and antiepileptic), were also obtained from the electronic records after being reviewed and confirmed by a neurologist.

Part of the survey was an Arabic-validated version of the Generalized Anxiety Disorder-7 (GAD-7) questionnaire (17). The GAD-7 is a short self-administered scale ranging from 0 to 21, with 0–4 for minimal anxiety, 5–9 for mild anxiety, 10–14 for moderate anxiety, and 15–21 for severe anxiety. We used a cutoff score of 10 for the diagnosis of generalized anxiety disorder (18). The GAD-7 has shown improved internal validity and reliability in studies involving patients with MS (19).

The last part of the survey was an Arabic-validated version of the Patient Health Questionnaire-9 (PHQ-9) (20). The PHQ-9 is a short self-administered scale ranging from 0 to 27, with 0–4 for minimal depression, 5–9 for mild depression, 10–14 for moderate depression, 15–19 for moderately severe depression, and 20–27 for severe depression. With a cutoff score of >9, the scale has good sensitivity and specificity, making it an appropriate tool for depression screening in patients with MS (21).

### 2.3. Data analysis

Continuous variables were reported as mean and standard deviation. Categorical variables were reported as frequencies and percentages. Data were analyzed using the chi-square, Fisher's exact, and analysis of variance tests, as appropriate. In addition, Spearman's test was used for correlation analysis. Statistical significance was set at *p* < 0.05. The goodness of fit showed *p* < 0.001; however, the distribution of the variables displayed on the central limit theorem was just slightly skewed. Therefore, mean and SD were still valid based on the central limit theorem to describe the center and variation of the reported continuous variables. Statistical analyses were performed using JMP statistical software subsidiary, SAS Institute, version 15.2.0.

## 3. Results

A total of 192 patients were included in this study. The mean age of the participants was 34.4 (±9.9) years. Most of the participants were women (68.2%), whereas 31.8% were men. Nearly half of the patients were married (48.9%), and 44.8% were employed. The majority of participants were non-smokers (75%). More than half of the patients were diagnosed with MS for >5 years (59.9%), with RRMS being the most common type (94.2%). The most frequently reported medications for MS were ocrelizumab (59.3%) and interferon beta (16.2%). Most patients (81.3%) had an EDSS score of < 6. The additional demographic details are presented in Table 1.

**Table 1:** Demographic characteristics of participants (*n* = 192).

**Item**	
Age, mean (SD)	34.4 (±9.9)
**Sex**, ***N*** **(%)**	
Male	61 (31.8%)
Female	131 (68.2%)
**Marital status**, ***N*** **(%)**	
Married	94 (49%)
Unmarried	81 (42.2%)
Divorced	13 (6.8%)
Widow	4 (2.1%)
**Occupation**, ***N*** **(%)**	
Employed	86 (44.8%)
Unemployed	78 (40.6%)
Retired	8 (4.2%)
Student	20 (10.4%)
**Monthly income**, ***N*** **(%)**	
< 3,000 Riyal	76 (39.6%)
3,000–6,000 Riyal	31 (16.1%)
6,000–9,000 Riyal	37 (19.3%)
>10,000 Riyal	48 (25%)
**Smoking**, ***N*** **(%)**	
No	144 (75%)
Yes	36 (18.8%)
Previous smoker	12 (6.2%)
**Year of diagnosis**, ***N*** **(%)**	
≤ 5 years	77 (40.1%)
>5 years	115 (59.9%)
**MS type**, ***N*** **(%)**	
CIS	1 (0.5%)
PPMS	4 (2.1%)
RRMS	181 (94.3%)
SPMS	6 (3.1%)
**Currently taking MS medications**	167 (87%)
Ocrelizumab	99 (59.3%)
Interferon beta	27 (16.2%)
Fingolimod	19 (11.4%)
Natalizumab	18 (10.7%)
Others	24 (14.4%)
Not taking MS medication	25 (13%)
**History of psychiatric disorders**, ***N*** **(%)**	
Yes	28 (14.6%)
No	164 (85.4%)
**EDSS score**	
< 6	143 (81.3%)
≥6	33 (18.7%)

**Figure 1:**
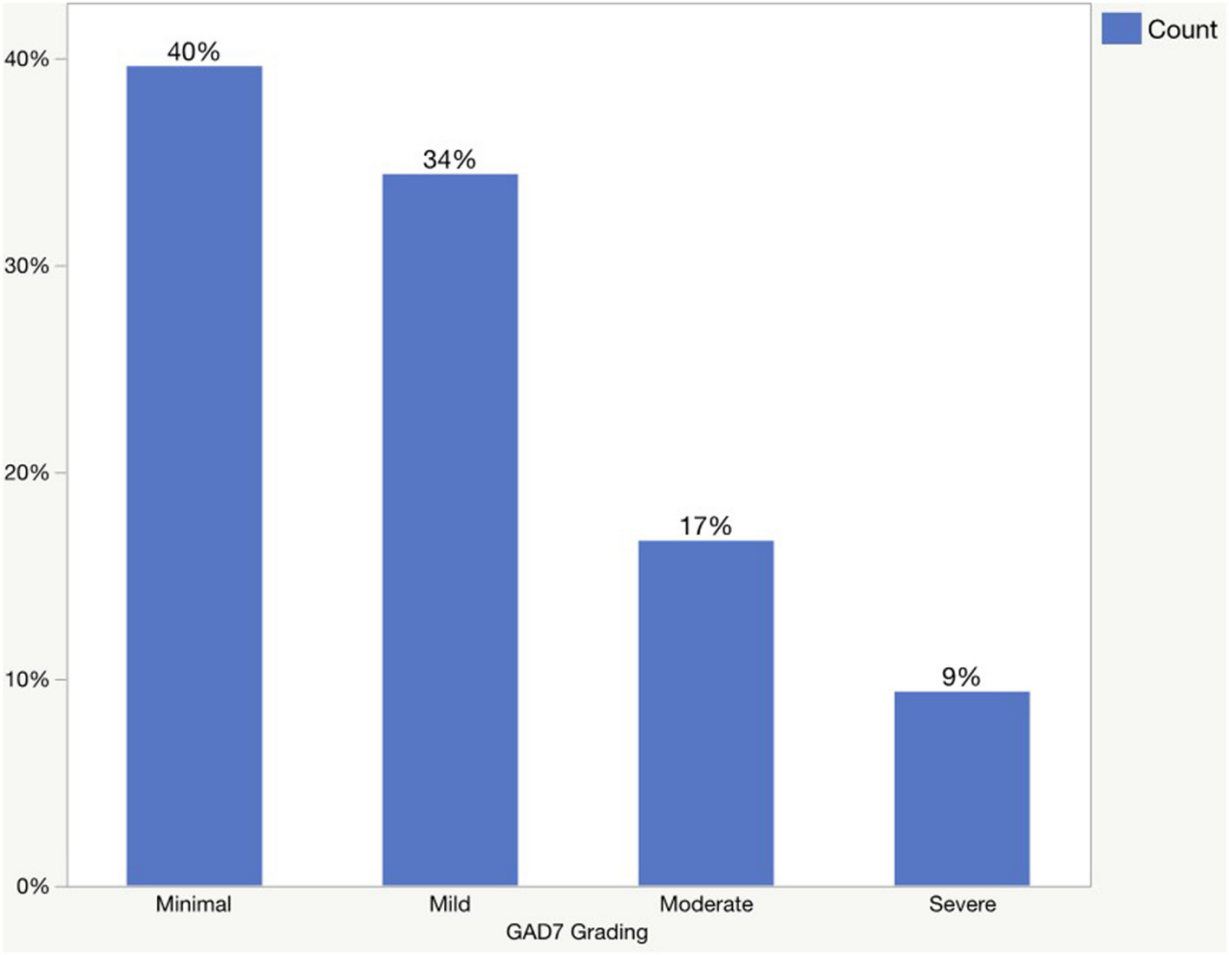
The prevalence of generalized anxiety disorder based on GAD-7 scores.

**Figure 2:**
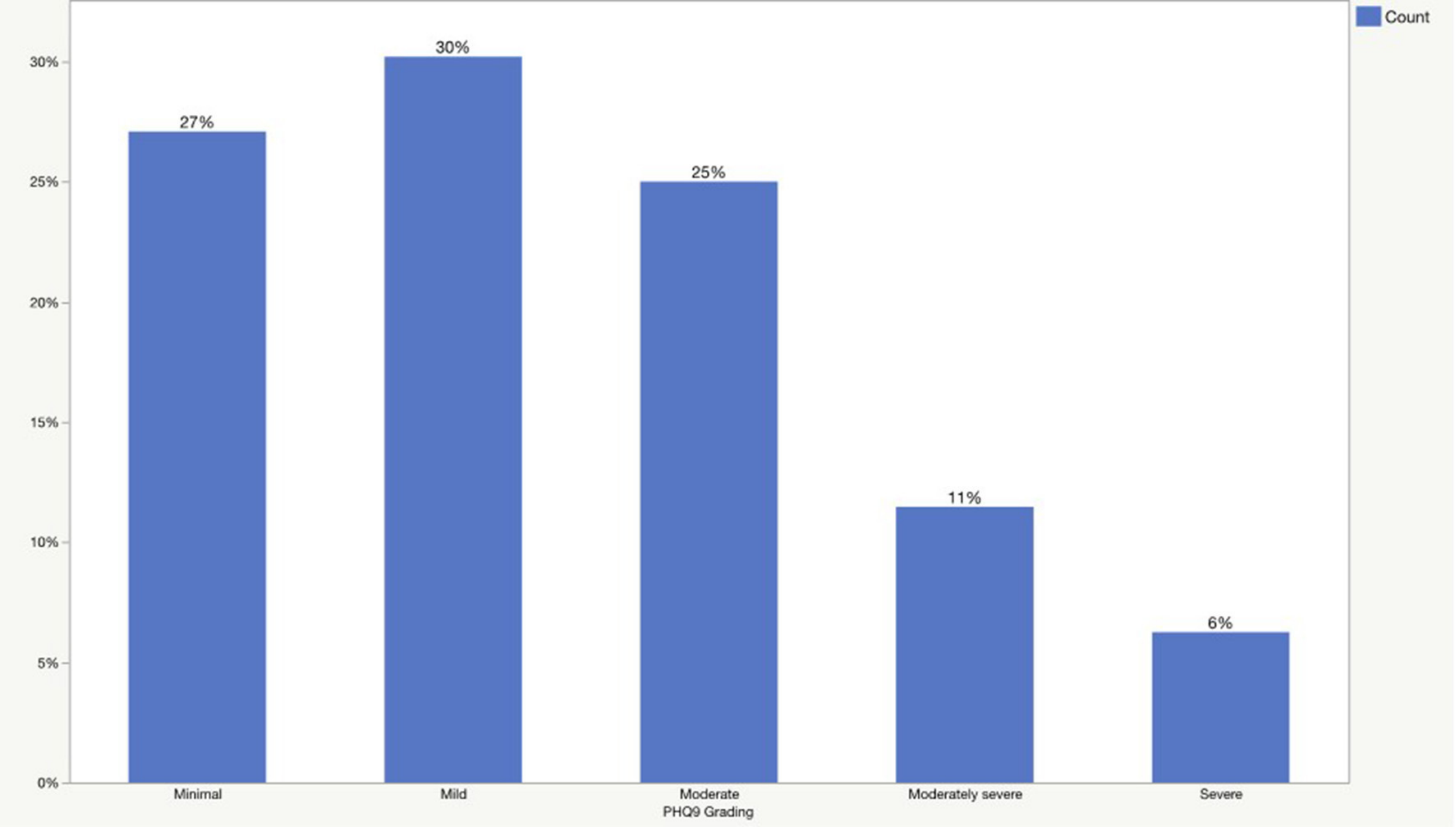
The prevalence of major depression based on PHQ-9 scores.

The prevalence of generalized anxiety disorder and major depression is presented in Figures 1, 2. According to the GAD-7 score, 40% of patients had minimal anxiety, followed by mild (34%), moderate (17%), and severe (9%) anxiety. Regarding the PHQ-9 scores, 27% of the patients had a minimal form of depression, followed by mild (30%), moderate (25%), moderately severe (11%), and severe (6%) depression.

Overall, the mean score of GAD-7 was 6.5 (±4.9), and generalized anxiety disorder was present in 26% (*n* = 50) of the participants. Meanwhile, the mean score of PHQ-9 was 8.8 (±6.1), and major depression was detected in 42.7% (*n* = 80) of the participants (Table 2).

**Table 2:** The prevalence of generalized anxiety disorder and major depression.

**Items**	
**GAD-7 score, mean (SD)**	6.5 (±4.9)
GAD-7, *N* (%)	
Positive screen for anxiety (≥10)^*^	50 (26%)
Negative screen for anxiety (< 10)^*^	142 (74%)
**PHQ-9 score, mean (SD)**	8.8 (±6.1)
PHQ-9, *N* (%)	
Positive screen for depression (≥10)^*^	82 (42.7%)
Negative screen for depression (< 10)^*^	110 (57.3%)

^*^Based on cutoff score of 10 in GAD-7 or PHQ-9.

Based on the GAD-7 score, 40 (80%) of those who met the criteria for generalized anxiety disorder were women, whereas only 10 (20%) were men, with a statistically significant difference (*p* = 0.0376). With respect to other demographic variables, no significant associations were found between those with anxiety and those without. However, patients with anxiety had more previous psychiatric disorders than patients without anxiety (*p* = 0.0283). More details are shown in Table 3.

**Table 3:** The association between the GAD-7 grading and patients' demographics, medical history, and multiple sclerosis-related variables.

	**Positive screen for generalized anxiety disorder (*n =* 50)**	**Negative screen for generalized anxiety disorder (*n =* 142)**	***p*-value**
**Sex**, ***N*** **(%)**			
Male	10 (20%)	51 (35.9%)	0.0376
Female	40 (80%)	91 (64.1%)	
**Marital status**, ***N*** **(%)**			
Married	28 (56%)	66 (46.5%)	0.6049
Unmarried	19 (38%)	62 (43.7%)	
Divorced	3 (6%)	10 (7%)	
Widow	0 (0)	4 (2.8%)	
**Occupation**, ***N*** **(%)**			
Employed	23 (46%)	63 (44.4%)	
Unemployed	19 (38%)	59 (41.5%)	0.8748
Retired	3 (6%)	5 (3.5%)	
Student	5 (10%)	15 (10.6%)	
**Monthly income**, ***N*** **(%)**			
< 3,000 Riyal	16 (32%)	60 (42.2%)	0.2423
3,000–6,000 Riyal	12 (24%)	19 (13.4%)	
6,000–9,000 Riyal	8 (16%)	29 (20.4%)	
>10,000 Riyal	14 (28%)	34 (24%)	
**Smoking**, ***N*** **(%)**			
No	42 (84%)	102 (71.8%)	
Yes	7 (14%)	29 (20.4%)	0.1740
Previous smoker	1 (2%)	11 (7.8%)	
**Year of MS diagnosis**, ***N*** **(%)**			
≤ 5 Years	22 (44%)	55 (38.7%)	0.5134
>5 Years	28 (56%)	87 (61.3%)	
**Currently taking MS medications**, ***N*** **(%)**			
Yes	45 (90%)	122 (85.9%)	0.4605
No	5 (10%)	20 (14.1%)	
**History of psychiatric disease**, ***N*** **(%)**			
Yes	12 (24%)	16 (11.3%)	0.0283
No	38 (76%)	126 (88.7%)	
**MS type**, ***N*** **(%)**			
CIS	0 (0)	1 (0.7%)	
PPMS	1 (2%)	3 (2.1%)	0.5411
RRMS	49 (98%)	132 (93%)	
SPMS	0 (0)	6 (4.2%)	

Based on the PHQ-9 score, 61 (74.4%) of those who had major depression were women, whereas only 21 (25.6%) were men, with a non-statistically significant difference (*p* = 0.1134). With respect to other variables, neither demographic nor MS-related variables were found to be significantly associated with depression. The additional details are provided in Table 4.

**Table 4:** The association between the PHQ-9 grading and patients' demographics, medical history, and multiple sclerosis-related variables.

***N* (%)**	**Positive screen for depression (*n =* 82)**	**Negative screen for depression (*n =* 110)**	***p*-value**
**Sex**, ***N*** **(%)**			
Male	21 (25.6%)	40 (36.4%)	0.1134
Female	61 (74.4%)	70 (63.6%)	
**Marital status**, ***N*** **(%)**			
Married	39 (47.6%)	55 (50%)	
Unmarried	35 (42.7%)	46 (41.8%)	
Divorced	6 (7.3%)	7 (6.4%)	0.9751
Widow	2 (2.4%)	2 (1.8)	
**Occupation**, ***N*** **(%)**			
Employed	38 (46.4%)	48 (43.6%)	
Unemployed	32 (39%)	46 (41.8%)	0.6880
Retired	2 (2.4%)	6 (5.5%)	
Student	10 (12.2%)	10 (9.1%)	
**Monthly income in Saudi Riyal**, ***N*** **(%)**			
< 3,000	28 (34.2%)	48 (43.6%)	0.3624
3,000–6,000	17 (20.7%)	14 (12.7%)	
6,000–9,000	15 (18.3%)	22 (20%)	
>10,000	22 (26.8%)	26 (23.7%)	
**MS type**, ***N*** **(%)**			
CIS	0 (0)	1 (0.9%)	
PPMS	1 (1.2%)	3 (2.7%)	0.8207
RRMS	79 (96.3%)	102 (92.7%)	
SPMS	2 (2.5%)	4 (3.7%)	
**Smoking**, ***N*** **(%)**			
No	61 (74.4%)	83 (75.4%)	
Yes	17 (20.7%)	19 (17.3%)	0.6915
Previous smoker	4 (4.9%)	8 (7.3%)	
**Year of MS diagnosis**, ***N*** **(%)**			
≤ 5 years	34 (41.5%)	43 (39.1%)	0.7400
>5 years	48 (58.5%)	67 (60.9%)	
**Currently taking MS medications**, ***N*** **(%)**			
Yes	73 (89%)	94 (85.5%)	0.4672
No	9 (11%)	16 (14.5%)	
**History of psychiatric disease**, ***N*** **(%)**			
Yes	15 (18.3%)	13 (11.8%)	0.2086
No	67 (81.7%)	97 (88.2%)	

The results showed a positive correlation between the EDSS score and age (*r* = 0.4264, *p* < 0.0001). However, no correlation was found between functional disability (EDSS score) and the prevalence of anxiety and depression (Table 5).

**Table 5:** EDSS score correlation with GAD-7, PHQ-9, and age.

	**EDSS score (*r*-value)**	***p*-value**
GAD-7	0.0485	0.4367
PHQ-9	0.0842	0.2226
Age	0.4264	< 0.0001

## 4. Discussion

MS is a common neurodegenerative disease that affects the CNS, especially in young adults. A major concern among the population with MS is the high prevalence of psychiatric comorbidities and their association with disease progression. Previous studies have also explained the impact of depression in patients with MS, including no adherence to medications, poor quality of life, deteriorating disability, and impaired cognitive function (22–25). Although the pathogenesis remains unclear, some studies suggest that demyelinating lesions in patients with MS contribute to their emotional disturbances (26, 27). In particular, lesions in the frontal lobe, prefrontal cortex, anterior temporal lobe, and parietal lobe can be associated with an increased incidence of anxiety and depressive disorders (10, 11). Therefore, patients with MS are more susceptible to psychiatric illnesses than the general population (9). This study aimed to determine the prevalence of major depression and generalized anxiety disorder among patients with MS in Saudi Arabia and its association with multiple demographic and MS-related variables.

Similar to the current literature, the majority of our study participants were women (68.2%), highlighting the fact that MS is more common in women (1). Based on the current study, the prevalence of generalized anxiety disorder and depression was 26% and 42.7%, respectively. This is consistent with a systematic review that found that anxiety and depression are the most reported psychiatric diseases, affecting more than 20% of patients with MS (28). Similar to our study, another systematic review found the prevalence of depression and anxiety to be 30.5 and 22.1%, respectively (9). However, the prevalence of depression and anxiety in this study is much higher compared to the general population in Saudi Arabia as the Saudi National Mental Health Survey (SNMHS) estimated the prevalence to be 3.8% for depression and 12.3% for anxiety in the general population in Saudi Arabia (29). In addition, a minority of patients (14.5%) reported a positive history of psychiatric disorders, mostly depression and anxiety. This could point out several issues related to this population, including stigma about mental health seeking behavior and beliefs that MS does not have a role in these complaints.

The association between MS and psychiatric disorders is related to several factors, including age, sex, year of diagnosis, EDSS score, and socioeconomic status. In accordance with sex, the majority of patients with depression were women (74.4%, *n* = 61). These findings are compatible with a recently published local study of 238 patients with MS, which concluded a higher prevalence of depression among women (16). In contrast, some studies have found no sex difference in terms of depression (30–32). In addition, the prevalence of anxiety was high and more pronounced in women in this study, comprising 80% (*n* = 40) of those with anxiety. Similarly, this was evident in two systematic reviews, associating MS with a higher prevalence of anxiety (28, 33). In this study, we did not find a correlation between disease duration and prevalence of psychiatric disorders. However, other studies suggested that shorter disease duration is a risk factor for anxiety only but not depression (13, 34).

In terms of monthly income, 39.4% (*n* = 76) of the participants had a low income (< 3,000 riyals). Furthermore, a significant proportion (40.6%, *n* = 78) of the participants were unemployed. Although unemployment could partially explain low income, it can be an MS sequela. One study found that patients with MS were more likely to be jobless or have changed careers to less skilled jobs as a consequence of their disease (31).

A minority of our sample, 14.6% (*n* = 28), reported a history of psychiatric disorder, and it was associated with a higher prevalence of anxiety only but not depression. Although both conditions can co-exist and their clinical pictures may overlap, anxiety symptoms are common and occur in 26–63.4% of patients with MS (28). This fact, in addition to the low number of patients with positive psychiatric history in our sample, could explain the association with anxiety and not depression.

Although our data reported a correlation between the EDSS score and age (*p* < 0.0001), no correlation was found between the EDSS and the GAD-7 or PHQ-9 scales. Similarly, a Norwegian study concluded that the EDSS score was not associated with psychiatric disorders (35). On the other hand, Alsaadi et al. reported a significant association between the EDSS scores and mood disorders (36). One possible explanation for this finding is that the EDSS score focuses mainly on physical disability and motor system dysfunction, rather than on direct relation to mood, cognitive, or psychiatric manifestation (37).

The results of this study must be considered within the context of certain limitations. Owing to the limited number of patients with MS visiting the clinic, the sample size of the study was small. However, this was compensated for by optimizing the quality of the data collected for each patient; therefore, it can be as representative as possible. Subsequent studies should include more participants and a larger sample to assess more demographic as well as MS-related variables, such as different MS phenotypes, which was a limitation in the present study. Moreover, the study relied on PHQ-9 and GAD-7, which are self-administered assessment tools, and the definitive diagnosis can only be made on clinical grounds with a full psychiatric assessment of the patients. Furthermore, a clinical diagnostic interview, such as the MINI International Neuropsychiatric Interview, might be considered in future studies. In addition, patients with MS have a higher chance of experiencing depression and anxiety; therefore, clinicians should routinely screen for these psychiatric disorders.

In conclusion, the current study demonstrated a higher prevalence of major depression and generalized anxiety disorder in patients with MS than in the general population. Female patients generally scored higher on the PHQ-9 and GAD-7 scales than male patients. No correlation was found between the EDSS score and disease duration with regard to the risk of developing depression and anxiety. As depression and anxiety are prevalent comorbidities in patients with MS and could affect the disease course negatively, screening and early intervention are crucial.

## Data availability statement

The raw data supporting the conclusions of this article will be made available by the authors, without undue reservation.

## Ethics statement

The studies involving human participants were reviewed and approved by the Institutional Review Board, King Abdullah International Medical Research Center, Riyadh, Saudi Arabia. The patients/participants provided their written informed consent to participate in this study.

## Author contributions

AMA, BA, ARA, and OB were responsible for the design and initial conception of the study. ARA, OB, EA, JA, AAl, and OW participated in data acquisition and assembly. BA and AMA provided statistical expertise and carried out the analysis of the data. AA, YA, AAb, and SM critically revised the manuscript. All authors participated in initial drafting of the manuscript, reviewed, and approved the final draft of the manuscript.
